# High p-Smad2 expression in stromal fibroblasts predicts poor survival in patients with clinical stage I to IIIA non-small cell lung cancer

**DOI:** 10.1186/1477-7819-12-328

**Published:** 2014-11-05

**Authors:** Yongbing Chen, Pengfei Xing, Yuanyuan Chen, Li Zou, Yongsheng Zhang, Feng Li, Xueguan Lu

**Affiliations:** Department of Thoracic Surgery, the Second Affiliated Hospital of Soochow University, 1055 Sanxiang Road, Suzhou, 215004 Jiangsu Province P.R. China; Department of Oncology & Radiotherapy, the Second Affiliated Hospital of Soochow University, 1055 Sanxiang Road, Suzhou, 215004 Jiangsu Province P.R. China; Department of Pathology, the Second Affiliated Hospital of Soochow University, 1055 Sanxiang Road, Suzhou, 215004 Jiangsu Province P.R. China

**Keywords:** lung cancer, stromal fibroblasts, p-Smad2, prognosis

## Abstract

**Background:**

Increasing evidence indicates that the TGFβ/Smad signaling pathway plays a prominent role in tumor initiation, progression, and metastasis. Therefore, we investigate the expression of p-Smad2 in surgical resection specimens from non-small cell lung cancer, and evaluate the prognostic significance of p-Smad2 expression in stromal fibroblasts and cancer cells for patients with clinical stage I to IIIA non-small cell lung cancer.

**Methods:**

The immunohistochemical expression of p-Smad2 was evaluated in 78 formalin-fixed paraffin-embedded surgical resection specimens from clinical stage I to IIIA non-small cell lung cancer. Correlations between p-Smad2 expression and clinicopathologic characteristics were determined by Chi-square test. The prognostic significance of p-Smad2 expression in stromal fibroblasts and cancer cells with regard to overall survival was determined by Kaplan-Meier.

**Results:**

There were 38.5% (30/78) and 92.3% (72/78) patients with high p-Smad2 expression in stromal fibroblasts and cancer cells, respectively. There was a positive correlation between the p-Smad2 expression level in stromal fibroblasts and the p-Smad2 expression level in cancer cells (*χ*^2^ = 4.176, *P* =0.045). No significant correlation of p-Smad2 expression in stromal fibroblasts or cancer cells with any of clinicopathologic characteristics was found. The 3-year overall survival rates with low and high p-Smad2 expression in stromal fibroblasts were 53.7% and 37.7%, respectively (*χ*^2^ = 3.86, *P* = 0.049). No significant association was found between low and high p-Smad2 expression in cancer cells with respect to overall survival, respectively (*χ*^2^ = 0.34, *P* =0.562).

**Conclusions:**

The results suggested that high p-Smad2 expression in stromal fibroblasts predicted poor survival in patients with clinical stage I to IIIA non-small cell lung cancer.

## Background

Non-small cell lung cancer (NSCLC) accounts for 80% of all lung cancers and is the most common cause of cancer-related death worldwide [1, 2]. In spite of recent advances in surgical techniques, chemotherapy, radiotherapy and many kinds of new strategies of treatment, long-term survival is achieved in only 5 to 10% of NSCLC patients [3]. The molecular mechanisms involved in lung carcinogenesis are associated with dysregulation of many signaling pathways. Among these, transforming growth factor-β (TGF-β) signaling plays an important role in development and progression of lung cancer [4–6]. TGF-β displays a dual role in cancer development. In early tumorigenesis, it functions as a tumor suppressor, whereas in later stages of tumor progression, it acts as a tumor promoter [7]. TGF-β is synthesized as a latent, extracellular matrix (ECM)-bound molecule, which is activated via proteolytic and nonproteolytic pathways. Activated TGF-β binds to TGF-β type-II receptor (TβRII), which subsequently recruits and transphosphorylates the type ITGF-β receptor activin receptor-like kinase (ALK)-5. Subsequently, intracellular signaling is mediated by the family of Smad proteins comprising eight members (Smad [1–8]). TGF-β receptor-activated Smad2 and Smad3 form heteromeric complexes with the common mediator Smad4 and then translocate to the nucleus where they act as ligand-induced transcription regulators of target genes [8, 9].

In recent years, increasing evidence indicates that the progression of tumors toward a malignant phenotype does not only depend on the cell-autonomous properties of cancer cells themselves but is also deeply influenced by tumor stroma [10]. The activated stromal fibroblasts, termed cancer-associated fibroblasts (CAFs), play a prominent role in tumor initiation, progression, and metastasis [11, 12]. TGF-β is implicated in the activation of tumor stromal fibroblasts, leading to the generation of CAFs and to promoting tumor stromal formation through the TGFβ/Smad signaling pathway [13–15].

Several previous studies have demonstrated that Smad2 expression level in cancer cells appeared to be correlated with tumor development and prognosis in patients with gastric carcinoma, glioma, breast cancer, colorectal cancer, and esophageal squamous cell carcinoma [16, 17]. However, not much is known regarding the prognostic value of Smad2 expression in lung cancer cells. In addition, there is especially a lack of evidence about the prognostic significance of Smad2 expression in stromal fibroblasts of lung cancer. In the present study, therefore, we investigated the p-Smad2 expression in surgical resection specimens from NSCLC to evaluate the prognostic significance of p-Smad2 expression in stromal fibroblasts and cancer cells for patients with clinical stage I to IIIA NSCLC.

## Methods

### Patients and surgical resection specimen selection

The paraffin-embedded postoperative tumor specimens were obtained from the tissue bank in the Department of Pathology, the Second Affiliated Hospital of Soochow University, between January of 2009 and June of 2011. We retrospectively recruited 78 tumor specimens from patients with clinical stage I to IIIA NSCLC. The ethics committee of the Second Affiliated Hospital of Soochow University approved the current project, and waived the need for written informed consent.

The main characteristics of the 78 patients with clinical stage I to IIIA NSCLC were summarized in Table 1. Ages of the patients in this study range from 41 to 81 years (median, 63 years). According to the AJCC/UICC (6th edition), there were 20 patients with stage І, 9 patients with stage II, and 49 patients with stage IIIA. All patients underwent curative resection. Systemic adjuvant treatment was administered to 64 patients. The chemotherapeutic regimen used was a cisplatin-based doublet. Seventeen patients received thoracic postoperative radiotherapy.

**Table 1:** **Patient characteristics**

Characteristic	Number of patients (%)
Patients	78 (100.0)
Median age = 63 years (range 41 to 81)	
Gender	
Male	55 (70.5)
Female	23 (29.5)
Pathologic type	
Squamous carcinoma	41 (52.6)
Adenocarcinoma	29 (37.2)
Adenosquamous carcinoma	3 (3.8)
Large cell carcinoma	5 (6.4)
Pathologic differentiation	
High	5 (6.4)
Median	50 (64.1)
Low	23 (29.5)
Clinical Stage*	
I	20 (25.6)
II	9 (11.5)
IIIA	49 (62.9)
Treatment modality - Curative resection	
Yes	78 (100.0)
No	0 (0)
Treatment modality - Adjuvant chemotherapy	
Yes	64 (82.1)
No	14 (17.9)
Treatment modality - Postoperative radiotherapy	
Yes	17 (21.8)
No	61 (78.2)

*According to Union for International Cancer Control/American Joint Committee on Cancer (6th edition) stage system.

### Immunohistochemistry

Serial slides, each 3-um thick, were cut from paraffin-embedded tissue. One slide was stained with hematoxylin and eosin (HE). Immunohistochemical staining was performed on another slide, using the two-step procedure. The anti-human p-Smad2 rabbit polyclonal antibody (Ser465/467) (Chemicon, Billerica, MA, USA; diluted 1: 500) was used. After de-paraffinization and hydration, the slides were subjected to antigen retrieval by pressure-cooking for 30 minutes. Endogenous peroxidase activity was neutralized using peroxide block placement on the slides for 15 minutes at room temperature. The slides were then incubated with anti-p-Smad2 antibody for 40 minutes at 4°C. This was followed by incubation with peroxidase-conjugated polymer (ChemMate EnVision/HRP; Gene Tech, Shanghai, China) for 30 minutes at room temperature. The chromogen reaction was developed in 3,3′-diaminobenzidine (DAB; Gene Tech, Shanghai, China) tetrahydrochloride for 10 minutes. Finally, hematoxylin was used as a light nuclear counterstain. The negative control used was an IgG2b isotype antibody (Dako), ensuring the same concentration of immunoglobins as for anti-p-Smad2.

### Assessment of p-Smad2 expression

All slides were evaluated independently by two experienced pathologists (Li F and Zhang Y). Ten high-power fields were selected randomly for each slide. The expression levels of each marker in cancer cells and stromal fibroblasts were independently evaluated. The percentage of positive-staining cells were graded on a scale of 0to 3, with less than 5% positive-staining cells as grade 0, 5 to 25% as grade 1, 26 to 50% as grade 2, and more than 50% as grade 3. The intensity of staining was also graded on a scale of 0 to 2, with negative to weak intensity as grade 0, weak to moderate intensity as grade 1, and moderate to strong intensity as grade 2. After that, the percentage score was multiplied by the intensity score. A final score between 0 and 2 was defined as low expression, and a score higher than 2 was defined as high expression.

### Statistical analysis

The association between p-Smad2 expression in cancer cells or stromal fibroblasts and clincopathologic characteristics was examined with a Chi-square test. Overall survival (OS) rates were performed by the Kaplan-Meier method and log-rank test. Overall survival duration was defined from the day of surgery to the day of death or last follow-up. For all tests, a two-sided *P* <0.05 was considered significant.

## Results

### Expression of p-Smad2 in stromal fibroblasts and cancer cells

**Figure 1:**
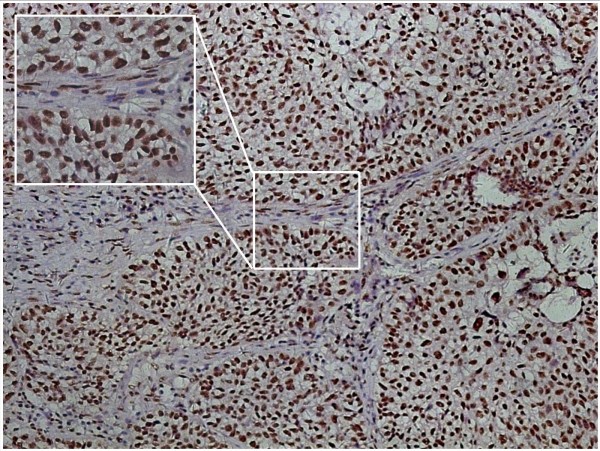
**Expression of p-Smad2 in stromal fibroblasts and in cancer cells by immunohistochemistry in non-small cell lung cancer (NSCLC) (magnification × 200).**

The expression of p-Smad2 was confined to the nucleus. The expression level of p-Smad2 in stromal fibroblasts ranged from 12.5% to 85.8%, and its expression level in cancer cells ranged from 19.3% to 94.2%. There were 38.5% (30/78) and 92.3% (72/78) patients with high p-Smad2 expression in stromal fibroblasts and cancer cells, respectively (Figure 1). The analysis revealed that there was a positive correlation between the p-Smad2 expression level in stromal fibroblasts and the p-Smad2 expression level in cancer cells (*χ*^2^ = 4.176, *P* = 0.045). With regard to age, gender, clinical stage, pathologic type and differentiation, there was no significant correlation of p-Smad2 expression in stromal fibroblasts or cancer cells with any of clinicopathologic characteristics (Table 2).

**Table 2:** **Association between p-Smad2 expression in cancer cells or stromal fibroblasts and clinicopathologic characteristics**

		p-Smad2 expression in cancer cells			p-Smad2 expression in stromal fibroblasts		
Variables	Number of patients	Low (%)	High (%)	*P*	Low (%)	High (%)	*P*
Gender							
Male	55	5 (9.1)	50 (90.9)	0.473	32 (58.2)	23 (41.8)	0.346
Female	23	1 (4.3)	22 (95.7)		16 (69.6)	7 (30.4)	
Age (years)							
≤63	41	2 (4.9)	39 (95.1)	0.326	23 (56.1)	18 (43.9)	0.298
>63	37	4 (10.8)	33 (89.2)		25 (67.6)	12 (32.4)	
Pathologic type							
Squamous carcinoma	41	2 (4.9)	39 (95.1)	0.560	24 (58.5)	17 (41.5)	0.605
Adenocarcinoma	29	3 (10.3)	26 (89.7)		20 (69.0)	9 (31.0)	
Adenosquamous carcinoma	3	0 (0)	3 (100.0)		2 (66.7)	1 (33.3)	
Large cell carcinoma	5	1 (20.0)	4 (80.0)		2 (40.0)	3 (60.0)	
Pathologic differentiation							
High	5	0 (0)	5 (100.0)	0.562	4 (80.0)	1 (20.0)	0.679
Median	50	5 (10.0)	45 (90.0)		30 (60.0)	20 (40.0)	
Low	23	1 (4.3)	22 (95.7)		14 (60.9)	9 (39.1)	
Clinical Stage*							
I + II	29	4 (13.8)	25 (86.2)	0.120	20 (69.0)	9 (31.0)	0.300
IIIA	49	2 (4.1)	47 (95.9)		28 (57.1)	21(42.9)	

*According to Union for International Cancer Control/American Joint Committee on Cancer (6th edition) stage system.

### Prognosis analysis

**Figure 2:**
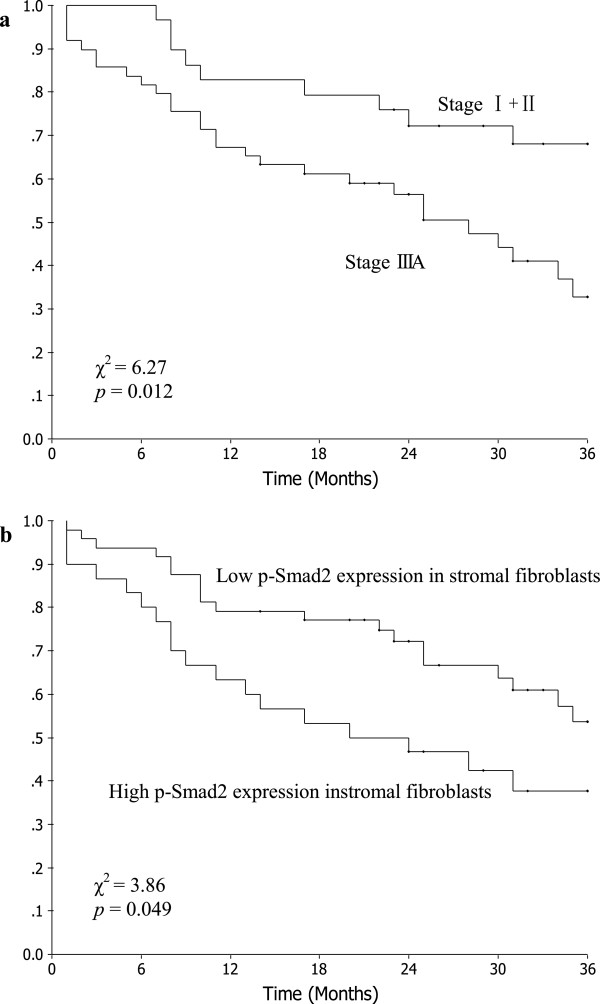
**Kaplan-Meier curves for patients with clinical stage I to IIIA non-small cell lung cancer (NSCLC). a**. different clinical stage groups; **b**. different p-Smad2 expression groups.

The median duration of follow-up for these patients was 26 months (range, 1 to 55 months). The Kaplan-Meier plots showed that the 3-year OS rate of all patients was 47.6%. The 3-year OS rates with clinical stage I + II and IIIA were 68.0% and 32.8%, respectively (*χ*^2^ = 6.27, *P* = 0.012; Figure 2a). The 3-year OS rates with low and high p-Smad2 expression in stromal fibroblasts were 53.7% and 37.7%, respectively (*χ*^2^ = 3.86, *P* = 0.049; Figure 2b). However, no significant association was found between low and high p-Smad2 expression in cancer cells with respect to OS, respectively (*χ*^2^ = 0.34, *P* = 0.562).

## Discussion

Because p-Smad2 is a primary step and intracellular signaling effector for the mediation of intracellular signaling of TGFβ [18], we focused our study on p-Smad2 expression on NSCLC. The present study demonstrated that p-Smad2 expression was confined to the nucleus, and its expression level in cancer cells was high in 72 (92.3%) of 78 NSCLC. However, Shinto *et al.* [16] found that the high expression level of p-Smad2 in cancer cells was existed in 63 (47%) of 135 gastric carcinomas. de Kruijf *et al.* [10] found that 129 (26.9%) of 505 breast cancers had high nuclear expression of p-Smad2 in cancer cells. These results suggest that the expression level of p-Smad2 in cancer cells is associated with tumor type. Recently, a few studies have demonstrated that Smad2 expression level in cancer cells is correlated with tumor development and prognosis. Previous studies on esophageal squamous cell carcinoma, breast cancer and colorectal cancer demonstrated that loss of Smad2 expression was correlated with tumor development and poor prognosis [19–21]. Conversely, Shinto *et al.* [16] found that the prognosis for p-Smad2-high patients with advanced stage gastric carcinoma was significantly poorer than that of p-Smad2-low patients. de Kruijf *et al.* [10] found that high expression of p-Smad2 was substantially associated with a worse prognosis in breast cancer. Similarly, the positive p-Smad2 expression was reported to be correlated with tumor proliferation and poor prognosis in a study of 52 patients with glioma [22]. At the same time, some studies demonstrated that p-Smad2 expression level was not related to the prognosis in patients with renal clear cell carcinoma and colorectal cancer [23, 24]. In the present study, we also found that the expression level of p-Smad2 in cancer cells was not associated with any of clinicopathologic characteristics and 3-year overall survival in patients with clinical stage I to IIIA NSCLC. These studies suggest that the results are inconsistent, even completely opposite in the same tumor type, and the further study is needed.

In recent years, increasing evidence indicates that activating TGFβ/Smad signaling pathway promotes the generation of CAFs and tumor stromal formation, and plays prominent roles in tumor initiation, progression, and metastasis [11–15]. So we further investigated p-Smad2 expression in stromal fibroblasts and its prognostic significance in NSCLC. The results found that 38.5% (30/78) patients with NSCLC had high p-Smad2 expression in stromal fibroblasts. Hawinkels *et al.* [9] found that 89% and 42% of 88 patients with colorectal cancer were observed with nuclear p-Smad2 expression in stromal fibroblasts and cancer cells. Moreover, we also found that there was a positive correlation between the p-Smad2 expression level in stromal fibroblasts and the p-Smad2 expression level in cancer cells (*P* = 0.045). The high p-Smad2 expression in stromal fibroblast was associated with a trend for poor overall survival in patients with clinical stage I to IIIA NSCLC. As we all know, this is the first report that focuses on prognostic significance of p-Smad2 expression in stromal fibroblasts.

## Conclusions

In conclusion, the results suggest that high p-Smad2 expression in stromal fibroblasts predicted poor survival in patients with clinical stage I to IIIA NSCLC. However, this study is limited by the relatively small number of patients, and more and larger studies are required.

## Abbreviations

ALK: activin receptor-like kinase
CAFs: cancer-associated fibroblasts
TβRII: TGF-β type-II receptor
NSCLC: non-small cell lung cancer
OS: overall survival
TGF-β: transforming growth factor-β.
